# Utilization of dual contraception method among reproductive age women on antiretroviral therapy in selected public hospitals of Northern Ethiopia

**DOI:** 10.1186/s12978-017-0390-6

**Published:** 2017-10-05

**Authors:** Solomon Weldemariam Gebrehiwot, Gedion Asnake Azeze, Carmen C. Robles, Yohannes Mehretie Adinew

**Affiliations:** 1 College of Health Sciences and Medicine, Wolaita Sodo University, Sodo, Ethiopia; 2 Department of Midwifery, College of Health Science, Mekelle University, P.O. Box 1871, Mekelle, Ethiopia

**Keywords:** HIV/AIDS, STIs, Dual contraceptive, Associated factors, Ethiopia

## Abstract

**Background:**

Sexually transmitted infections are highly prevalent among pregnant women in Africa. Among the incidence of HIV infection in children, 90% of the infection is attributable to their mothers. Ethiopia is one of the countries with an increasing risky sexual behavior and the most affected by the HIV epidemic. If prevention of mother to child transmission focuses on increasing contraception, it will prevent more than 29% of HIV infection at birth. Therefore, the aim of this study was to assess utilization of dual contraceptive  method and associated factors among reproductive age women on antiretroviral therapy in selected public hospitals of Mekelle town, Northern Ethiopia.

**Methods:**

Institution based cross-sectional survey was conducted in selected public hospitals of Mekelle among women under antiretroviral therapy from March 1–April 31, 2016. We used a systematic sampling technique to select 331 women. A pretested interviewer administered questionnaire was used for data collection. The data were entered in to Epi data version 3.1 and exported to SPSS version 20 for analysis. Bivariate and multivariable logistic regression analysis was computed. Odds ratio along with 95% CI was computed to ascertain the association. Statistical tests at *p*-value of < 0.05 were considered as cut off point to determine statistical significance.

**Results:**

Only 51(15.7%) of participants have utilized dual contraception method. Being single[AOR 5.43, 95% CI (1.61, 18.32)] and cohabitated [AOR 6.06; 95% CI: (2.16, 16.95)] in marital status, having HIV negative partner [AOR 4.44; 95% CI: (1.23, 16.04)], exposure to post diagnosis counseling [AOR 3.03; 95% CI: 1.34, 6.80], disclosed HIV status [AOR 6.06; 95% CI: (1.78, 20.87)] and discussing safer sex with partner [AOR 6.96; 95% CI: (2.75, 16.62)] were positively associated with utilization of dual contraceptive method.

**Conclusion:**

The overall magnitude of dual contraceptive use is still low in this study. This will be a great concern on the transmission of the virus from mother to babies and partners and risk of complications following unintended pregnancy. This will continue to present as major public health problems in the region unless future interventions focuses on the barriers through tailored counseling and husband involvement in all aspects of the HIV/AIDS care.

**Electronic supplementary material:**

The online version of this article (10.1186/s12978-017-0390-6) contains supplementary material, which is available to authorized users.

## Plain English summary

HIV positive mothers should give special emphasis in reproductive health care services since they are a special group in reproductive health care to maintain healthy generation in the future. Studies revealed that unintended pregnancy was significantly higher in HIV positive women (20.7%) as compared to their counterparts (13.5%). Therefore, the aim of this study is to explore the underlying associated factors with utilization of dual method contraception among reproductive age women on antiretroviral therapy. The study was conducted in two Hospitals from March 1 to 31, 2016. Systematic sampling technique was used to select study participants. The number of clients sampled from each hospital was proportional to the total number of client flow registered in the Antiretroviral Therapy (ART) unit. Data were collected by face to face interview technique using a structured questionnaire. Strength of association was determined using multivariable logistic regression model. Accordingly, Becoming single and cohabitated marital status, HIV status of partner, post diagnosis counseling, disclosure status and discussion with partner regarding safer sex were significantly associated factors. Hence, strengthening the counseling session and encouraging husband involvement during counseling sessions about contraception and STIs prevention strategies are highly recommended.

## Background

More than 2 million HIV positive women become pregnant every year due to poor contraceptive utilization and unsafe sex practices, among them, 600,000 die of obstetric complications [1, 2].

Women who are on Highly Active Antiretroviral Therapy (HAART) and uses only highly effective contraceptive methods without condom, this circumstances will double the risk of acquisition of drug resistant strains of HIV-1 infection and twice as likely to pass the infection to their partners [3, 4]. Although the increasing availability and use of HAART service has improved quality of life in people living with HIV (PLHIV), high total fertility rate (4.8%), low contraceptive utilization (29%) and significant proportion of mother to child transmission (MTCT) (30%) were among the risk factors for HIV infection increment [5].

Studies revealed that unintended pregnancy was significantly higher in HIV positive women (20.7%) as compared to their counterparts (13.5%) [6]. Among the incidence of HIV infection in children, 90% of cases are attributable to mother to child transmission (MTCT) [7]. If prevention of mother to child transmission (PMTCT) focuses on increasing contraception, it will prevent more than 29% of HIV infection at birth [8]. By dual protection is a protection against both risks of unintended pregnancy and sexually transmitted infections (STIs) including HIV [9, 10]. This dual objective could be achieved by using condom alone or the use of condom plus another effective contraceptive; like injectables, implants, pills, intrauterine contraceptive device(IUCD) (for those clinically stable or on Anti-Retroviral Therapy(ART)) and tubal sterilization in sexually active couples [11]. Moreover, it is also important to reduce pediatric HIV and obstetric complications following unintended pregnancy [3, 12–15]. Theoretically, dual protection can be accomplished by consistent use of male/female condom alone; however condom use alone as contraceptive method does not substantially decrease risk of pregnancy and in general it is not reliable method for women on ART [16, 17]. It can only prevent up to 85% of unintended pregnancy [11, 18]. On the other hand hormonal contraceptives, sterilization and intrauterine contraceptive device (IUCD) are effective in preventing unintended pregnancy as compared to condom but cannot prevent STIs including HIV [16, 18]. The effectiveness of low-dose hormonal contraceptives may be reduced by ART drugs which necessitates the use of condom to compensate this gap [3]. Therefore, the best approach to dual protection is use of dual contraceptive methods [11].

Despite the obvious advantages, the United Nation Program on HIV/AIDS (UNAIDS) 2013, report has shown Ethiopia is one of the countries with an increasing risky sexual behavior and is most affected by the HIV epidemic [19]. Utilization of dual method contraception has been reported to be very low from research settings [3, 20]. Prevention of unintended pregnancy and sexual transmitted infections (STIs) including new strains of HIV infections among HIV positive women using condom plus another highly effective method is one of the ‘cornerstones of a comprehensive program for PMTCT [21]. As far as our knowledge, many of the studies conducted previously in Ethiopia focused on contraceptive method utilization however studies on dual contraceptive method use were limited. The finding may contribute to increase knowledge and evidence on dual contraception method utilization in the country setting. Moreover, the results will be useful to public health policy makers and practitioners to restructure the service delivery system and formulate programs to reach the target group who are in need of dual method use. Therefore, the objective of this study was to assess utilization of dual contraception method and associated factors among reproductive age women on Anti Retroviral Therapy (ART) in selected public hospitals of Mekelle town, Northern Ethiopia.

## Methods

### Study setting and design

The study was conducted in two selected public hospitals of Mekelle town (Ayder comprehensive specialized hospital and Mekelle general hospital) the capital city of Tigray regional state located at the northern part of Ethiopia, about 783 km from Addis Ababa the capital city of Ethiopia. The major reason to consider these two hospitals as study area were, most people living with HIV/AIDS in Mekelle are attending at these two hospitals and they are the largest by’ providing services from Mekelle town. In Ethiopia PMTCT related care is free of charge in public health facilities and most of the women prefer them as they can’t afford the private ones. In addition, Ayder is a well equipped referral hospital for the entire Tigray region. However, the rest two public hospitals (Quiha, Semien Eiz hospital) and other public health centers have limited flow of patients. Moreover, a budget constraint was also another reason. Ayder comprehensive specialized hospital ART unit has given service for a total of 1117 clients since 2009, and Mekelle General Hospital has given service to 8669 ART users since 2003. Institution based cross sectional study was employed from March 1 to 31, 2016.

### Study population and sampling procedures

The study population includes all HIV positive reproductive age women enrolled on ART units of the two hospitals. Women who were reproductive age group and sexually active in the past 3 months were eligible for this study. Pregnant women and women who were incapable to fertilize or reproduce for different reasons excluded from this study. A single population proportion formula [n = (z α/2)^2^ p(1-p) /d^2^] was used to calculate the sample size with the assumption of (26.7%) proportion of dual contraception use [22], 95% confidence level, 5% margin of error and 10% of non response rate. Overall, we recruited a total of 331 (223 respondents from Ayder referral hospital and 108 from Mekelle general hospital) respondents. The number of clients sampled from each hospital was proportional to the total number of eligible clients flow registered in the ART unit in 1 month by taking 3 months average of client flow. On average per a month, there were 354 eligible ART clients flow in Ayder comprehensive specialized hospital and 728 in Mekelle general hospital. We used systematic sampling technique to recruit every k^th^ eligible respondents and we used sequence/registration of client flow at outpatient department (OPD) as sampling frame. The value of “k” was fortunately 3 for both hospitals. Lottery method was used to select the first participant by preparing 1 to 3 ranges of numbers then every subsequent participant was selected by skipping 2 clients until we fill the required sample size. Dual contraception method was defined as the use of male/female condom along with other highly effective contraceptive methods like pills, injectable, implants, sterilization (male/female) and intrauterine contraceptive device (IUCD) consistently in the last 3 months prior to the study. Use of condom alone as contraceptive method may not be effective to prevent unintended pregnancy. On the other hand, the other highly effective contraceptive methods are effective in preventing unintended pregnancy however they cannot prevent STIs including HIV. Moreover, condom is the only barrier method that can prevent STIs including HIV. Therefore, condom was taken as constant with other highly effective contraceptive methods.

### Data collection tool and procedures

The questionnaire was adapted from published literatures and translated in to Tigrigna language (local language) then back to English language to check internal consistency. Four pre-service nursing students were collected the data by face to face interview technique using a structured questionnaire. A pre-test was conducted among 5% (17 clients) of ART clients in Wukro hospital outside of Mekelle but with in similar set up. Appropriate modifications were made after analyzing the pretest result before the actual data collection. Data quality was assured by giving training and appropriate supervision for data collectors. Data collectors and supervisor were local language (Tigrigna) speakers. The overall supervision was carried out by the principal investigator and supervisors. The collected data were also cross checked on each day for its consistency and completeness.

### Data processing and analysis

The collected data were checked for completeness and entered into Epi Data software version 3.1 and exported to SPSS window version 20 for data processing and analysis. Descriptive statistics like percent, mean and standard deviation were done. Both bivariate and multivariable logistic regression analysis were computed to identify associated factors. Odds ratio along with 95% CI was computed to ascertain the association between independent and outcome variables. Variables that have *p*-value of <0.2 at bivariate analysis were included in multivariable logistic regression to control possible confounding factors. Statistical tests at p-value of <0.05 were considered as cut off point to determine statistical significance.

### Ethical consideration

Ethical clearance was obtained from the institutional ethical review board of Mekelle University, College of Health Sciences. Official letter of permission was written to the respective hospitals by Tigray Regional Health Office to Mekelle hospital and Medical director of the hospital to ART unit of Ayder referral hospital. Participants were informed about the purpose, benefit, risk, confidentiality of information and the voluntary nature of participation in the study. The interview was conducted in a private room. Data were collected after informed written consent was obtained from each participant that their interview data will be included in publications. For participants less than 18 years old, consent was taken from their parents/Guardian.

## Results

### Socio-demographic characteristics of respondents

Among the 331 sampled eligible respondents, 324 of them responded to the questionnaire completely, yielding a response rate of 97.9%. The 7 non-responders were due to refusal to participate four and missed pages by the data collector during interview three. The mean age of the respondents was 32.49 years (SD ± 6.21). Of the total respondents, majority of them were urban residents, 281(86.7%), Tigrian in ethnicity, 269(83%), followed by Amhara, 40(12.3%), and others (Oromo and Afar), 15(4.7%) (Table 1).

**Table 1:** Socio demographic variables of HIV positive women on ART in public Hospitals of Mekelle town, Ethiopia 2016

Variables	Frequency (n)	Percent (%)
Age		
15–19	9	2.9
20–24	21	6.5
25–29	61	18.8
30–39	187	57.6
40–49	46	14.2
Marital status		
Married	194	59.9
Married but separated	34	10.5
Cohabitating	50	15.4
Single	46	14.2
Religion		
Orthodox	265	81.8
Muslim	51	15.7
Protestant	8	2.5
Occupation		
House wife	126	38.9
Merchant	64	19.7
Daily laborer	37	11.4
Government employee	59	18.2
Student	19	5.9
Others^a^	19	5.9
Women’s educational status		
Illiterate	88	27.2
Primary education	109	33.6
Secondary education	87	26.9
Tertiary education	40	12.3
Husband’s educational status		
Illiterate	45	13.8
Primary education	86	26.5
Secondary education	122	37.8
Tertiary education	71	21.9

Others^a^: Commercial sex workers, farmer and house servant

### Risk prevention behavior, access to information and reproductive characteristics

Nearly half 157(48.5%) of the women had history of condom use and 97(61.8%) of them were using it consistently. Reported reasons for not using condom were 74 (44.3%) partners’ refusal, 61(36.5%) perceiving it as a barrier to sexual pleasure and 43(25.7%) being considering it ineffective. Almost half (48.5%) of the respondents had one to two biologically alive child (median of 2.0 and IQR; 1, 3) (Table 2)**.**

**Table 2:** Risk prevention behavior, access to information and reproductive characteristics of HIV positive women on ART in public hospitals of Mekelle town, Ethiopia 2016

Variables *N* = 324	Frequency (N)	Percent (%)
Use of condom		
Yes	157	48.5
No	167	51.5
Consistency of condom use		
Consistent	97	61.8
Inconsistent	60	38.2
Reasons to use condom^a^		
To prevent STI/HIV	100	63.7
To prevent pregnancy	97	61.8
To prevent new HIV strain	69	43.9
Reason for not using condom^a^		
Refusal by partner	74	44.3
Interruption of sexual pleasure	61	36.5
Condoms ineffectiveness	43	25.7
Religion	42	25.1
Trust issues	29	17.4
Breakage/slippage	17	10.2
Fear of side effect	12	7.2
Lack of access	10	6.0
Post diagnosis counseling		
Yes	117	36.1
No/don’t remember	207	63.9
FP counseling by ART provider		
Yes	203	62.7
No/ don’t remember	121	37.3
Information where to get contraception		
Yes	225	69.4
No/ don’t remember	99	30.6
Prior experience of sexual violence		
Yes	78	24.1
No	246	75.9
Number of alive child/children		
None	59	18.2
1–2	157	48.5
3–4	70	21.6
≥ 5	38	11.7
Future desire for more children		
Yes	208	64.2
No	116	35.8

^a^Respondents were allowed for more than one answer

### HIV disclosure and decision making status characteristics

The participants median value of most recent CD4 count was 379 cells/dl (IQR; 261.50, 560.75 cells/dl). More than one third 125 (38.6%) of study participants did not know their husband’s/partner’s HIV status. Among those women who were aware of their partner’s HIV status, 42 (21.1%) were sero-discordant (Table 3).

**Table 3:** HIV disclosure and decision making status among HIV positive women on ART in public Hospitals of Mekelle town, Ethiopia, 2016

Variables (N = 324)	Frequency (n)	Percentage (%)
Most recent CD4 count		
< 250 cells/dl	74	22.9
250–350 cells/dl	73	22.5
> 350 cells/dl	177	54.6
Prior experience of TB infection		
Yes	89	27.5
No	235	72.5
Disclosure of HIV status		
Yes	205	63.3
No	119	36.7
To whom HIV disclosed?		
To spouse/sexual partner	102	31.5
To parents/family	79	24.4
To the community as voluntary	17	5.2
To friends	7	2.2
Reason for not disclosing HIV status		
Fear of stigma and discrimination	41	34.5
Lack of trust on people	29	24.3
Other	49	41.2
Decision maker on women’s sexual and RH		
Her self	171	52.8
together with husband	136	42.0
Husband alone	17	5.2
Free discussion with husband		
Yes	126	38.9
No	198	61.1

### Magnitude of dual contraceptive utilization

Only 51(15.74%) of women stated consistent use of dual contraception method. The most common form of dual method reported was condom combined with Injectable (68.6%), implants (19.6%), followed by pills (11.8%). But no respondent has used condom plus intrauterine contraceptive device (IUCD) or sterilization method. Among the effective contraceptive users, the highest percentage was accounted by, 113 (34.9%) Injectable, 54 (16.7%) pills and 39 (12%) implants.

### Factors associated with utilization of dual contraception method

After controlling the effect of confounding variables, marital status, HIV status, post diagnosis counseling, disclosure status and free discussion with partner regarding safer sex were significantly associated with dual contraceptive method. Accordingly, the odds of using dual contraceptive method was 5 [AOR 5.43, 95% CI (1.61, 18.32)] and 6 [AOR 6.06, CI (2.16, 16.95)] times higher among the single and cohabitated women respectively when compared to the married ones.

Partner’s HIV status was an important predictor of dual contraceptive utilization. The odds of using dual contraceptive methods were about 4 [AOR 4.44, 95% CI (1.23, 16.04)] times higher among women whose partners were HIV negative when compared to those whose partner’s status were unknown. Furthermore, the odds of using dual contraceptives methods were 3 [AOR 3.04, 95% CI (1.35, 6.80)] times higher among women who had received post diagnosis counseling compared to their counterparts.

Women who had no desire for more children were 2 [AOR 2.65, 95% CI (1.16, 6.07)] times more likely to use dual contraceptive method when compared to those who had desire. Compared to women who failed to disclose their HIV status, the odds of dual method use was 6 [AOR 6.08, 95% CI (1.77, 20.87)] times higher among those who disclosed their status. Open discussion between partners was also strong predictor of dual contraceptive utilization. The odds of using dual contraception among women who had free discussion with their husband about safer sex were nearly 7 [AOR 6.96, 95% CI (2.75, 17.62)] times higher as compared to those who had none (Table 4).

**Table 4:** Bivariate and multivariable logistic regression analysis of factors associated with dual contraception utilization among women on ART in public hospitals of northern, Ethiopia 2016

Variables	Dual method utilization		Crude OR (95% CI)	Adjusted OR (95% CI)
	No (%)	Yes (%)		
Age				
15–19	7 (77.8)	2 (22.2)	1	
20–24	18 (85.7)	3 (14.3)	0.58 (0.08, 4.27)	
25–29	43 (70.5)	18 (29.5)	1.46 (0.27, 7.74)	
30–39	160 (85.6)	27 (14.4)	0.59 (0.11, 2.99)	
40–49	45 (97.8)	1 (2.2)	0.07 (0.01, 0.97)	
Marital status				
Married	173 (89.2)	21 (10.8)	1	1
Single	33 (71.7)	13 (28.3)	3.24 (1.47, 7.11)	5.43 (1.61, 18.32)*
Married but separated/	32 (94.1)	2 (5.9)	0.51 (0.11, 2.30)	1.12 (0.19, 6.46)
Cohabitating	35 (70.0)	15 (30.0)	3.53 (1.65, 7.51)	6.06 (2.16, 16.95)*
Women’s education status				
No formal education	81 (92.0)	7 (8.0)	1	
Primary education	100 (91.7)	9 (8.3)	1.04 (0.37, 2.91)	
Secondary education	65 (74.7)	22 (25.3)	3.91 (1.57, 9.74)	
Tertiary and above	27 (67.5)	13 (32.5)	5.57 (2.01, 15.40)	
Husband’s HIV status				
Unknown	115 (92.0)	10 (8.0)	1 2.49	1
HIV positive	129 (82.2)	28 (17.8)	(1.16, 5.36)	0.72 (0.22, 2.33)
HIV negative	29 (69.0)	13 (31.0)	5.15 (2.05, 12.93)	4.44 (1.23, 16.04)*
Post diagnosis counseling				
No	189 (91.3)	18 (8.7)	1	1
Yes	84 (71.8)	33 (28.2)	4.12 (2.19, 7.73)	3.03 (1.35, 6.8)*
Number of alive child				
None	47 (79.7)	12 (20.3)	1	
1–2	127 (80.9)	30 (19.1)	0.925 (0.48, 1.95)	
3–4	64 (91.4)	6 (8.6)	0.367 (0.12, 1.04)	
>/=5	35 (92.1)	3 (7.9)	0.336 (0.08, 1.28)	
Future desire for more child				
No	90 (77.6)	26 (22.4)	2.11 (1.15, 3.86)	2.65 (1.16, 6.07)*
Yes	183 (88.0)	25 (12.0)	1	1
Disclosure of HIV status				
No	113 (95.0)	6 (5.0)	1	1
Yes	160 (78.0)	45 (22.0)	5.29 (2.19, 12.84)	6.08 (1.77, 20.87)*
Decision maker on women’s sexual and RH				
The respondent alone	152 (88.9)	19 (11.1)	1	
The husband alone	15 (88.2)	2 (11.8)	1.06 (0.22, 5.02)	
Together	106 (77.9)	30 (22.1)	2.26 (1.21, 4.23)	
Free discussion				
No	189 (95.5)	9 (4.5)	1	1
Yes	84 (66.7)	42 (33.3)	10.5 (4.88,22.55)	6.95(2.74, 17.61)*

*Significantly associated at *p* value of <0.05

## Discussion

The uptake of dual contraceptive method was found to be very low. Marital status, HIV status, post diagnosis counseling, disclosure status and discussion with partner regarding safer sex were significantly associated factors with utilization of dual contraceptive methods.

Dual method contraceptive utilization in the study area was found to be 51(15.7%) which is very low. This result is consistent with the findings from different parts of Ethiopia (Tigray region (14%) [7], Addis Ababa (14.7%) [8]), and South-Africa (14.4%) [23]. However, the current finding is higher as compared to previous reports from rural Uganda and USA, where 3.5% and 7.5% of women used dual contraceptive method respectively [24, 25]. This might be due to an intervention that has been taking place in Ethiopia within the time gap since the time of the previous studies reported, where awareness creation about dual contraceptive method utilization is being advocated in Ethiopia. Currently, the government is expanding the family planning service and condom distribution including quality of service which might have increased dual contraceptive utilization.

On the contrary, the magnitude of dual method use in this study was lower when compared to studies conducted in Fitche, Ethiopia (32%) [26], Southeast Nigeria (27.2%) [27] and India (23%) [11].The possible explanation for this difference might be partly due to the socio-demographic difference; plus the study from India includes men in their interview while the current study uses only women as study population. Men may have over reported non-barrier contraceptive use among their wives because the wives might not directly control condom use. Moreover, respondents from the India study were volunteer peer educators, thus, there might be sampling bias by recruiting more people educated about HIV and contraceptive use. Being single and is cohabiting increased the likelihood of utilizing dual contraception method as compared to married women. This result is consistent with findings from Ethiopia [28] and United state [25] which revealed that married women more practiced dual method less than single women. This might be due to the fact that husbands may resist use of condoms in marital and steady relationships because sex ought to be natural and based on trust. Furthermore, in Ethiopia people associate condom use as a method for preventing STIs and acknowledges the potential for infidelity and distrust within relationship and they perceived that having sex with their husband is a low risk for transmission [29]. This could limit the uptake of dual methods.

Women whose partners were HIV negative were more likely to use dual contraception method when compared to women whose partners’ status were unknown. The finding is consistent with the findings from study conducted in India and France, where women whose partners were HIV negative were more likely to use dual contraceptive method as compared to their counter parts [11, 30]. This might be due to the fact that those women who knows their partners’ status are likely to be in committed relationships, therefore, able to negotiate condom use, and they might be more concerned for their sexual partners’ health to use dual methods.

Receiving post diagnosis counseling was also predictor of dual contraception use. Respondents who received post diagnosis counseling were more likely to use dual contraceptive method compared to their counterparts. This finding is in line with study from India [11] that reported receiving post diagnosis counseling as a significant factor. This could be explained by the assumption that women could get advice on the importance of condom use in addition to other effective contraceptives, how to negotiate with sexual partner and risk reduction strategies.

Women who had no desire for more children showed better use of dual contraceptive method than those who had desire. The finding is in line with findings from Fitche, Ethiopia [26] and South Africa [8]. The possible reason is that fertility desire is obviously a proximate factor for dual method use. Moreover, these HIV positive women might realize that the probability of vertical transmission to their baby and the probability of obstetrical complications associated with pregnancy and delivery.

Respondents who disclosed their HIV status used dual contraceptive method more as compared to those who failed to disclose. This finding was supported with a study done in Addis Abeba, Ethiopia and Ghana [20, 28]. It could be due to the fact that, women who disclose their status to their sexual partner are expected to be more counseled and knowledgeable on safer sex practices which will help them to have free discussion in regard to sexual matters [31]. But, those who failed to disclose their status might not insist on condom use as they might be scared of exposing the secret they are hiding.

Another finding of the present study is that women who had free discussion with their husband showed better use of dual contraceptive method than their counterparts. The result is in agreement with the studies conducted in Tigray [32] and Addis Ababa Ethiopia [28]. This might be because these women are expected to have more freedom to negotiate safer sex and birth spacing. Similarly, positive effects of open discussion on couples contraception use have been widely demonstrated in different studies [22, 33].

## Conclusions and recommendations

In spite of the fact that near half of the respondents in this study had history of condom use, the overall magnitude of dual contraceptive use is still low. It is an alarming message for developing countries; particularly to Ethiopia among the countries most affected by the HIV epidemic. This will be a great concern on the transmission of the virus from mother to babies, partners and obstetrical consequences following unintended pregnancy. This will continue to present as major public health problems in the region unless future interventions targeting on sexual activities and desire to have children. Hence, the efforts to increase dual method use should focus on strengthening the integration of family planning and HIV care service and encouraging tailored counseling and supportive care in the HIV/AIDS chronic care unit. Moreover, emphasis should be given to husband involvement in aspects of the HIV/AIDS care and disclosure status.

### Limitation of this study

Despite the contribution of the study to provide knowledge and evidence of dual contraceptive use in PLHIV, this study has some limitations to be considered.

First, it is a cross-sectional study in which temporal relations could not be assessed.

Since this study is institutional based, the result of this study may not be generalizable to mothers attending health institutions outside the study area and found at community level. Furthermore, social desirability and stigma may have biased respondents and may not be generalizable to others found to be HIV positive women.

## Additional file

Additional file 1: Dataset supporting data. (SAV 26 kb)

## Abbreviations

AIDS: Acquired immune deficiency syndrome
ART: Anti retroviral therapy
HAART: Highly active anti retroviral therapy
HIV: Human immune deficiency virus
IUCD: Intra uterine contraceptive device
MTCT: Mother to child transmission
PMTCT: Prevention of mother to child transmission
STI: Sexual transmitted disease
UNAIDS: United Nation program on HIV/AIDS
